# Understanding Contextual Factors in Cost, Quality and Priority Setting Decisions in Health: Comment on "Contextual Factors Influencing Cost and Quality Decisions in Health and Care: A Structured Evidence Review and Narrative Synthesis"

**DOI:** 10.15171/ijhpm.2018.82

**Published:** 2018-09-08

**Authors:** Stuart Peacock, Colene Bentley

**Affiliations:** ^1^Canadian Centre for Applied Research in Cancer Control (ARCC), Vancouver, BC, Canada.; ^2^BC Cancer, Vancouver, BC, Canada.; ^3^Faculty of Health Sciences, Simon Fraser University, Burnaby, BC, Canada.

**Keywords:** Healthcare Decision-Making, Cost, Quality, Health Management, Priority Setting

## Abstract

There is growing recognition in the academic literature that critical decisions concerning resource allocation and resource management in health and care are influenced by a range of contextual factors. In their paper in this journal, Williams et al define these ‘decisions of value’ as being characterized by a significant and demonstrable impact on quality and resources in health and care. ‘Decisions of value’ are key functions of health and care organizations, yet relatively little is known about how contextual factors (such as different sources and types of evidence used, organizational context and decision-making structures, and the wider interests of patients, the public and politicians) influence those decisions. In this commentary we offer some reflections on our international experiences in capacity building, developing and implementing priority setting and resource allocation (PSRA) mechanisms in the health and care sectors in a range of low-, middle-, and high-income countries. We focus on the role of organizational culture, the relationship to government including political and regulatory environments, and the potential for patient and public engagement in PSRA mechanisms.

There is growing recognition in the academic literature that critical decisions concerning resource allocation and resource management in health and care are influenced by a range of contextual factors. In their paper in *this journal*, Williams et al define these ‘decisions of value’ as being characterized by a significant and demonstrable impact on quality and resources in health and care.^1^ ‘Decisions of value’ are key functions of health and care organizations, yet relatively little is known about how contextual factors (such as different sources and types of evidence used, organizational context and decision-making structures, and the wider interests of patients, the public and politicians) influence those decisions.

For the last 25 years or so, much of our own work has focused on building capacity in, and developing more rational and fair mechanisms for priority setting and resource allocation (PSRA) decisions in health and care at the meso and macro/national levels. Although one of us trained as an economist, it seemed (at least to us) quite evident from very early on that decisions relating to value, quality and cost in health and care did not conform to the rigid and reductionist economists’ notion of ‘rational’ and evidence-based decision-making. Instead, information on effectiveness and cost-effectiveness seemed to be one important input in decision-making processes, but there were many other important (and legitimate) influences on those processes. To better understand these influences, one needs to takes a wider view using lenses from a range of disciplines, including but not limited to psychology, management, ethics and political economy. Many of these influences fall under the umbrella of ‘contextual factors.’

Williams et al make a strong addition to the literature on understanding how decisions relating to value in health and care are made in the real world. They do this by synthesizing the literature and providing an explanatory narrative around a range of contextual factors that influence decisions relating to value, cost and quality. They start by drawing a distinction between allocative decision-making processes (ie, those relating to the distribution of resources between alternative interventions and programmes) and technical decision-making processes (ie, those relating to investments made in order to enhance organizational capacity and functioning). Following Pettigrew, they characterize factors in terms of inner context (factors from within the organization) and outer context (factors external to the organization).^2^ The findings of their review highlight some interesting clusters of factors under each of these two broad groups. From the inner context, they identify sources of information (often clinical, ethical, and cost information), interests within the organization (including patients), organizational and institutional characteristics, governance and leadership and organizational culture. From the outer context, they identify external interests such as regulators, the media, and the public, economic factors, and relationship to government.

Several of the points the authors raise are particularly important, given our experiences in the development and implementation of PSRA mechanisms in the health and care sectors. First, the authors draw a distinction between ‘organizational and institutional characteristics’ and ‘organizational culture.’ We think this is a useful distinction to draw, and the authors are suitably circumspect about coming up with a clear definition of ‘organizational culture.’ Second, they discuss how the relationship to government including political and regulatory environments, can engender decision-making that is driven by compliance and risk aversion rather than outcomes. One only needs to spend a very short period of time in the health system to see the powerful influence of compliance and risk aversion in decisions. Third, the authors conclude that where decisions affecting costs and quality are of significant scale and scope there is a strong normative case for involving patients and citizens. This is a point with which we agree, and has been a research focus of significant interest in the last decade.

In what follows, we would like to offer some reflections on each of these three points. We have chosen to focus on these three points as they are particularly important in PSRA and allocative decision-making, as that is where our experience lies. These reflections draw on experiences we and international colleagues have had in developing and implementing PSRA decision processes in a number of countries over the last 25 years, employing methods from economics, ethics and decision sciences.

An important message from our experiences in developing and implementing PSRA mechanisms is the need for health economists and decision analysts to be practical and not expect organizations, managers, and clinicians to simply take up ‘rational’ and evidence-based approaches to priority setting. For a PSRA approach to be effective, analysts and decision-makers also need to consider a range of pragmatic and ethical considerations.^3,4^ Pragmatic considerations reflect the practical challenges that managers and clinicians face in allocating resources, including establishing organisational objectives, understanding organisational context and ensuring organisational readiness, and ascertaining that the implementation of results is feasible.^3,4^

In our experience perhaps the strongest challenge to any ‘rational’ and evidence-based approach to priority setting is that of organisational context and behaviour. Addressing issues such as the need for *relative* organisational stability (noting that flux is the norm rather than the exception in health services), the active involvement of high-level champions, an organisational culture that is receptive to fostering change, and an openness to learning are all critical factors to the success of evidence-based PSRA tools as practical decision-making aids. Effective management and leadership within health services is required to drive any PSRA process. The application of new priority-setting methods results in significant changes in decision-making culture, and potentially in the configuration of health and care services. At the same time, ownership by key stakeholders is necessary, including ownership by service managers/providers, and community/patient representatives. Providers will only own the process if they are involved from the outset and the process is internally driven. Similarly, there needs to be confidence that the chosen PSRA model will remain in place for some time and will be actively implemented.^3,4^

Our own experiences with PSRA processes seem to have a lot in common with the findings of Williams et al, namely, that organizational culture is hard to define, it can be unpacked to a degree, and it intersects with a number of other contextual factors, including organizational characteristics. One potential way forward may come from the literature on high-performing health organizations,^5^ which Smith et al have employed to develop a framework of high performance in PSRA based on four domains of structures, processes, attitudes and behaviours, and outcomes.^6^

Turning to the second point, once again it only takes a short period of time working in health and care systems to understand that the political environment can lead to risk aversion in many areas of decision-making. This is perhaps most evident in coverage and disinvestment decisions. Many decision-makers involved in the adoption and coverage decisions of new interventions will have experienced a political *volte face* on priority-setting decisions, where (for example) the Minister and her/his team reverse a decision to limit access to a new intervention. This is perhaps most evident in decisions relating to access to drugs for rare diseases and cancer drugs for patients with last-stage disease. This is not a criticism as such, since decision reversals typically reflect the democratic political systems in which they are made, and the reality is that politicians are sensitive to their electorate and other interest groups and therefore may reverse ‘unpopular’ decisions. The same applies to disinvestment recommendations. Indeed, both decision-makers and politicians are typically highly risk averse to disinvestment decisions, even when ‘rational’ and evidence-based processes show an intervention is either of no value or is of low value and those resources could be redirected to interventions that provide greater value to the community. Indeed, in our experiences it seems quite a stretch to suggest that decision-makers and politicians value losses in the same way they value gains; it is quite evident that disinvestment decisions are often treated fundamentally differently from adoption and coverage decisions.

Finally, to the third point: the normative case for involving patients and citizens. The last decade or so has seen a rapid increase in the development of methods and applications of both patient and public engagement in decisions of value in health and care. Our own interests lie mainly in deliberative public engagement, which we would suggest should have a growing role in determining what the most important consequences are of a funding decision. From a normative perspective, there is agreement among many stakeholders that public engagement is necessary to realize the democratic ideals of legitimacy, transparency, and accountability.^7^ Furthermore, these ideals have been advanced as necessary for supporting fair and ethical healthcare decision-making. On a more pragmatic level, public engagement can help stakeholders understand the degree of popular support for policy options, and may enhance public trust in decision-making processes. One-way consultative methods such as focus groups or surveys have traditionally been used to elicit public preferences in these frameworks. However, deliberative forms of public engagement are emerging as a viable policy-informing approach to civic participation in health and care decision-making. Specifically, deliberative public engagement involves members of the public in a process of learning and exchanging views explicitly directed towards collective problem-solving, thus making it distinct from other discussion-based consultation forums, like focus groups.^8^ We would suggest that deliberative public engagement methods have significant potential to inform ‘decisions of value’ in health and care. In particular, public engagement enhances accountability, may improve the legitimacy of decisions taken and can be used to work through moral conflict by creating platforms for shared decision-making on key policy issues.^9^

Lastly, we would like to return to the influence of the political environment on decision-making and in particular on risk aversion. We have both had the privilege of teaching in a number of professional graduate programs in health administration and policy over the years. We have always found these courses to be immensely enjoyable, and have learnt a tremendous amount from students and other faculty about how decisions really play out in health and care systems. Indeed, we would recommend any academic health economist to audit one of these courses to gain a good understanding of how uni-dimensional the ‘rational’ health economics model of PSRA can be, and how many other contextual factors shape how decisions relating to value should be, and are, made.

## Ethical issues

Not applicable.

## Competing interests

Authors declare that they have no competing interests.

## Authors’ contributions

Both authors jointly conceptualized and wrote this commentary.

## Authors’ affiliations

^1^Canadian Centre for Applied Research in Cancer Control (ARCC), Vancouver, BC, Canada. ^2^BC Cancer, Vancouver, BC, Canada. ^3^Faculty of Health Sciences, Simon Fraser University, Burnaby, BC, Canada.

## References

[1] Williams I, Brown H, Healy P (2018). Contextual factors influencing cost and quality decisions in health and care: a structured evidence review and narrative synthesis. Int J Health Policy Manag.

[2] Pettigrew A. The Awakening Giant: Continuity and Change in Imperial Chemical Industries. Chichester: Wiley Blackwell; 1985.

[3] Mitton C, Donaldson C. Priority Setting Toolkit: Guide to the Use of Economics in Healthcare Decision Making. London: BMJ Books; 2004.

[4] Peacock S, Ruta D, Mitton C, Donaldson C, Bate A, Murtagh M (2006). Using economics to set pragmatic and ethical priorities. BMJ.

[5] Baker GR, Denis JL. A Comparative Study of Three Transformative Healthcare Systems: Lessons for Canada. Ottawa, Ontario: Canadian Health Services Research Foundation; 2011.

[6] Smith N, Mitton C, Hall W (2016). High performance in healthcare priority setting and resource allocation: A literature- and case study-based framework in the Canadian context. Soc Sci Med.

[7] Abelson J, Wagner F, DeJean D (2016). Public And Patient Involvement In Health Technology Assessment: A Framework For Action. Int J Technol Assess Health Care.

[8] O’Doherty KC (2013). Synthesising the outputs of deliberation: Extracting meaningful results from a public forum. Journal of Public Deliberation.

[9] O’Doherty KC, Burgess MM (2009). Engaging the public on biobanks: outcomes of the BC biobank deliberation. Public Health Genomics.

